# Local and Systemic Interleukin-32 in Esophageal, Gastric, and Colorectal Cancers: Clinical and Diagnostic Significance

**DOI:** 10.3390/diagnostics10100785

**Published:** 2020-10-04

**Authors:** Dorota Diakowska, Małgorzata Krzystek-Korpacka

**Affiliations:** 1 Department of Gastrointestinal and General Surgery, Wroclaw Medical University, 50-368 Wroclaw, Poland; dorota.diakowska@umed.wroc.pl; 2 Department of Nervous System Diseases, Wroclaw Medical University, 51-618 Wroclaw, Poland; 3 Department of Medical Biochemistry, Wroclaw Medical University, 50-368 Wroclaw, Poland

**Keywords:** inflammation, epithelial-mesenchymal transition, cancer biomarker, anatomical subsite heterogeneity, immunomodulator, metastasis, angiogenesis, invasion, hypoxia, tumor molecular margin

## Abstract

Little is known on clinical and diagnostic relevance of interleukin-32 in gastrointestinal tract (GIT) cancers. We determined its mRNA (*n* = 52) and protein (*n* = 63) expression in paired (tumor-normal) samples from esophageal squamous cell carcinoma (ESCC) and gastric (GC) and colorectal cancer (CRC) patients, with reference to cancer-associated genes, and quantified circulating interleukin-32 in 70 cancer patients and 28 controls. *IL32* expression was significantly upregulated solely in ESCC, reflecting T stage in non-transformed tumor-adjacent tissue. Fold-change in *IL32* and IL-32 was higher in left-sided CRC, owing to high interleukin expression in non-transformed right-sided colonic mucosa. *IL32* was independently and positively associated with *Ki67*, *HIF1A*, and *ACTA2* and negatively with *TJP1* in tumors and with *IL10Ra* and *BCLxL* in non-transformed tumor-adjacent tissue. IL-32 protein was significantly upregulated in colorectal tumors. In ESCC, advanced stage and lymph node metastasis were associated with significant IL-32 upregulation. Circulating interleukin was significantly elevated in cancer patients, more so in ESCC and GC than CRC. As biomarker, IL-32 detected gastroesophageal cancers with 99.5% accuracy. In conclusion, IL-32 is upregulated in GIT cancers at local and systemic level, reflecting hypoxia and proliferative and invasive/metastatic capacity in tumors and immunosuppressive and antiapoptotic potential in non-transformed mucosa, while being an accurate biomarker of gastroesophageal cancers.

## 1. Introduction

Gastrointestinal tract (GIT) cancers, encompassing adenocarcinomas of the colorectum (CRC) and stomach (GC) and esophageal squamous cell carcinoma (ESCC), are the most common and deadliest malignancies [1]. Radical surgery, alone or in combination with chemotherapy or radiation, remains the main therapeutic option. Mortality rates are tightly associated with cancer stage at presentation. However, the GIT cancers, ESCC and GC in particular, are frequently diagnosed when advanced, rendering them non-amenable for curative resection. Available therapeutic options fail to improve outcomes for patients with gross metastatic disease and cancers resistant to chemo- and radiotherapy [2,3,4,5]. There is a growing awareness that in order to improve prognosis, a better understanding of cancer-associated abnormalities at molecular level is urgently needed to facilitate biomarker discovery and develop and implement patient-tailored approach, referred to as “precision medicine” [6,7,8,9].

Chronic inflammation with accompanying oxidative stress plays a crucial role in initiation of neoplastic transformation in GC and CRC [3,10]. Infection with *H. pylori* is a main risk factor in non-cardia GC, accounting for up-to 90% of cases. Other GC risk factors include infection with Epstein–Barr virus or autoimmune gastritis and thus are inflammation-related as well. Global attempt for *H. pylori* eradication managed to significantly reduce incidence of this subtype. However, it also contributed to substantial increase in cardia GC incidence. Cardia GC may have common etiology with non-cardia subtype or be associated with obesity and gastroesophageal reflux, conditions of persistent low-grade inflammation and oxidative stress [3]. Crohn’s disease and ulcerative colitis are two main phenotypes of inflammatory bowel disease (IBD), chronic relapsing condition, currently incurable, associated with increased risk for CRC [10]. While esophageal adenocarcinoma is a typical inflammation-associated cancer [11], the contribution of chronic inflammation to ESCC is more subtle. Overuse of strong alcohol and prolonged exposure to tobacco smoke, irritants and carcinogens inducing oxidative and genotoxic stress and evoking inflammation, are dominant risk factors only in some regions. Disturbed oral microbiome, infections with human papillomavirus, and improper diet—low on antioxidants and contaminated with nitrosamines or mycotoxins—are other increasingly recognized and inflammation- and oxidative stress-associated risk factors [4]. Noteworthy, in addition to its role in cancer initiation, inflammatory milieu supports tumor growth by providing mitogens and pro-survival cues and allows cancer cells to evade immune system and disseminate [12].

Interleukin (IL)-32 is a relatively recently discovered cytokine of potent pro-inflammatory activity. It is expressed in natural killer cells, monocytes, lymphocytes T, peripheral blood mononuclear cells, epithelial and endothelial cells, and fibroblasts in a number of isoforms with varying biological activity. Its expression is triggered by IL-1β, IL-18, tumor necrosis factor (TNF)-α, and interferon (IFN)-γ. IL-32 is engaged in setting an inflammatory loop as it in turn induces the synthesis of IL-1β, TNFα, IL-6, IL-8, and macrophage inflammatory protein (MIP)-2 [13,14,15]. Consistently, the involvement of IL-32 has been documented in infectious diseases, chronic inflammatory conditions, including gastritis and inflammatory bowel disease, and in cancer [13,14,15,16,17,18]. Amassing evidence indicates that the biological activity of IL-32 is cell type- and context-depended and displays isoform-specific nuances. Consequently, it may either facilitate or hamper cancer development, gaining IL-32 a catching label of “frenemy in cancer” [15]. Our view on the interleukin shifted from a simple inflammation amplifier to a modulator of inflammatory response and cell fate. Importantly, IL-32, as well as mechanisms employed in regulating formation of its endogenous isoforms, is considered a potential target for anti-neoplastic strategy [13,14,15,16].

Limited reports regarding GIT cancers show that the interleukin aids invasiveness of gastric cancer [19] but may act as a tumor suppressor in the colon [20]. In view of existing controversies and growing interest in the cytokine as potential anti-neoplastic target, the relative scarcity of data is surprising. Therefore, we aimed at comparative analysis of its local expression patterns in ESCC, GC, and CRC and at an appraisal of diagnostic power of circulating IL-32. The interleukin in the present study was quantified at mRNA and protein level and referred to cancer pathology and the local expression of a panel of cancer-associated genes (*IL4*, *IL4Ra*, *IL7*, *IL7Ra*, *IL10*, *IL10Ra*, *IL13*, *IL13Ra*, *ACTA2*, *BCL2*, *BCLxL*, *CCL2*, *CDKN1A*, *CLDN2*, *SLC2A1*, *HIF1A*, *Ki67*, *NOS2*, *ODC1*, *PTGS2*, *TJP1*, and *VEGFA*) as well as circulating cytokines and growth factors (IL-1β, IL-4, IL-6, IL-8, IL-12p70, fibroblast growth factor (FGF)-2, granulocyte colony-stimulating factor (G-CSF), granulocyte-macrophage colony-stimulating factor (GM-CSF), monocyte chemoattractant protein (MCP)-1, MIP-1α, platelet-derived growth factor (PDGF)-BB, TNFα, and vascular endothelial growth factor (VEGF)-A).

## 2. Materials and Methods

### 2.1. Study Population

Study population consisted of 100 individuals: 28 controls and 72 cancer patients with histologically confirmed esophageal squamous cell carcinoma (*n* = 17), gastric adenocarcinoma (*n* = 14), or colorectal adenocarcinoma (*n* = 41). Details are given in Table 1. Cancer patients were admitted to the Department of Gastrointestinal and General Surgery (Wroclaw Medical University) for curative tumor resection. Patients with any severe systemic illness or gross metastatic disease non-amenable for curative resection or subjected to previous radio- or chemotherapy were excluded. Enrolled patients underwent standard preoperative evaluation consisting of blood work, physical examination, and imaging (ultrasonography, computed tomography and magnetic resonance). Cancers were rated pathologically using 7th edition of the Union for International Cancer Control TNM system. In all examined cases, the resection margins were cancer-free. Serum samples from apparently healthy individuals were obtained from blood donors from the Regional Center of Blood Donation and Therapeutics in Wroclaw, Poland. Information on sample availability for transcriptional analysis and determination of local and systemic IL-32 protein concentration is given in Results in respective subsections.

### 2.2. Ethical Considerations

The study gained the acceptance of the Medical Ethics Committee of Wroclaw Medical University (#KB 203/2016 from 21 April 2016). It was conducted in accordance with the Helsinki Declaration of 1975, as revised in 1983, and informed consent was obtained from all study participants.

### 2.3. Analytical Methods

#### 2.3.1. *IL32* Expression in Tissue Samples

Pairs of tissue samples from the tumor and from the macroscopically normal tissue adjacent to the tumor (taken approximately 10 cm from the tumor) were taken postoperatively and rinsed with PBS. Samples were then immersed in RNAlater purchased from Ambion Inc. (Austin, TX, USA). Solution-soaked tissue samples were then stored at −80 °C until RNA isolation.

Tissue samples of 30–40 mg were homogenized in FastPrep-24 Homogenizer from MP Biomedical (Solon, OH, USA) using lysis buffer and 2-mercaptoethanol (100:1, *v*/*v*) from Sigma-Aldrich (St. Luis, MO, USA). The RNA was isolated using phenol-chloroform extraction and then purified with PureLink™ RNA Mini Kit (Invitrogen, Carlsbad, CA, USA). Genomic DNA was removed by on-column incubation with DNase (PureLink™ DNase Set, Invitrogen). The RNA isolates were quantified using NanoDrop 2000 from Thermo-Fisher Scientific (Waltham, MA, USA). Purity of isolated RNA was evaluated using 260/280 nm and 260/230 nm absorbance ratios. Its integrity was determined using Experion RNA StdSens analysis kits and the Experion platform, employing LabChip microfluidic technology (BioRad, Hercules, CA, USA). Aliquots of RNA isolates corresponding with 1000 ng per reaction mixture (20 µL) were reversely transcribed using iScript™ cDNA Synthesis Kit (BioRad) and C1000 termocycler (BioRad). reaction conditions were set as suggested by the manufacturer. Quantitative (real-time) polymerase chain reaction (qPCR) was conducted using SsoFast EvaGreen^®^ Supermix (BioRad) and CFX96 Real-Time PCR thermocycler (BioRad). The following cycling conditions were applied: 30 s activation at 95 °C, 5 s denaturation at 95 °C, annealing/extension for 5 s at 61 °C, 45 cycles, followed by melting step (60–95 °C with fluorescent reading every 0.5°C) to assure product specificity, further confirmed in an electrophoresis in high-resolution agarose (SeaKem LE agarose from Lonza, Basel, Switzerland) in TBE with SYBR Green (Lonza) detection. Reaction mixture consisted of cDNA (2 µL; diluted 1:5), 2×SsoFast EvaGreen^®^ Supermix (10 µL), 10 nM forward and reverse target-specific primers (1 µL of each), and water up to 20 µL. The following primer sequences were used: 5′-TCAAAGAGGGCTACCTGGAGAC-3′ (*IL32*, forward); 5′-TCTGTTGCCTCGGCACCGTAAT-3′ (*IL32*, reverse); 5′-TAGATTATTCTCTGATTTGGTCGTATTGG-3′ (*GAPDH*, forward); 5′-GCTCCTGGAAGATGGTGATGG-3′ (*GAPDH*, reverse). Primer sequences were synthesized by Genomed (Warsaw, Poland). Technical replicates were averaged prior analysis. Geometric mean of all Cq values in a given analysis was obtained and subtracted from sample Cq (ΔCq) then linearized by 2^ΔCq conversion and normalized to *GAPDH* (internal control). The obtained values were referred to as a normalized relative quantity (NRQ) [21] and subjected to statistical analysis.

Data on relative expression of *IL4*, *IL4Ra*, *IL7*, *IL7Ra*, *IL10*, *IL10Ra*, *IL13*, *IL13Ra*, *ACTA2*, *BCL2*, *BCLxL*, *CCL2*, *CDKN1A*, *CLDN2*, *SLC2A1*, *HIF1A*, *Ki67*, *NOS2*, *ODC1*, *PTGS2*, *TJP1*, and *VEGFA* in tissue samples investigated were available for 45 cancer patients and were retrieved from our earlier studies [22,23] for the purpose of correlation analysis.

#### 2.3.2. IL-32 Concentration in Tissue Homogenates

Pairs of tissue samples from the tumor and from the macroscopically normal tissue adjacent to the tumor (taken approximately 10 cm from the tumor) were taken postoperatively and rinsed with PBS. Samples were then rapidly frozen and stored at –45 °C until analysis.

Tissue fragments of 10–40 mg were placed in 10 mM Tris-HCl buffer with addition of 150 mM KCl and 1 mM EDTA (pH 7.4) in proportion 1:2 (*w/v*) and homogenized with ceramic spheres for 2 min at 4.0 m/s in FastPrep-24 homogenizer from MP Biomedical (Solon, OH, USA). Resulting homogenates were centrifuged (14,500× *g*, 10 min, 6 °C). Supernatants collected and used for IL-32 quantification using Human Interleukin 32 ELISA Kits from MyBiosource, Inc. (San Diego, CA, USA) according to manufacturer’s instructions. The assays were performed in duplicates and absorbance was measured using Microplate Reader BioTek ELx800 TS and Gen5 program (BioTek Instrument Inc., Winooski, VT, USA). Technical replicates were averaged and normalized to tissue weight and results are expressed as ng of protein per gram of analyzed tissue [ng/g].

#### 2.3.3. IL-32 Concentration in Serum Samples

Peripheral blood was drawn into BD Vacutainer CAT tubes (Becton Dickinson, Plymouth, UK) and clotted at room temperature for 30 min. Subsequently, samples were centrifuged (1500× *g* for 10 min at room temperature). Resulting serum samples were aliquoted and stored at −45 °C until examination. Blood was drawn upon patient’s admission, prior to any treatment and following overnight fast. For IL-32 quantification the same immunoassays as described above were used. Results are expressed as pg of interleukin per milliliter of serum [pg/mL].

Data on serum concentration of IL-1β, IL-4, IL-6, IL-8, IL-12p70, FGF2, G-CSF, GM-CSF, MCP-1, MIP-1α, PDGF-BB, TNFα, and VEGF-A, determined using Luminex xMAP technology, were available for 43 cancer patients and were retrieved from our earlier studies [24] for the purpose of correlation analysis.

### 2.4. Statistical Analysis

Data were tested for homogeneity of variances and normality of distribution using Levene test and Kolmogorov-Smirnov test, respectively. Pair-wise analysis was conducted using *t*-test for paired samples on log-transformed data (transcriptional analysis) or Wilcoxon test (protein determination). Two-group comparisons were conducted using *t*-test for independent samples, with Welch correction in case of unequal variances, or Mann–Whitney *U* test. Multigroup comparisons were conducted using one-way ANOVA with Tukey–Kramer post-hoc test or Kruskal–Wallis *H* test with Conover post-hoc test. Data were presented as geometric means with 95% confidence interval or medians. Correlation analysis was conducted using Spearman’s rank correlation test (*ρ*) or Pearson correlation (*r*). Frequency analysis was conducted using *χ*^2^ test. Receiver operating characteristics (ROC) curve analysis was conducted to determine the diagnostic power of IL-32. The overall marker accuracy was expressed as area under the ROC curve (AUC). In addition, marker sensitivity and specificity at optimal cut-off value were calculated. Multiple regression (backward and forward stepwise method) was applied to identify independent predictors of *IL32* expression. Their correlation with *IL32* after the influence of the remaining variables is eliminated is presented as partial correlation coefficients (r_p_).

All calculated probabilities were two-tailed. The *p* values ≤0.05 were considered statistically significant. The entire analysis was conducted using MedCalc Statistical Software version 19.4.0 (MedCalc Software Ltd., Ostend, Belgium; https://www.medcalc.org; 2020).

## 3. Results

### 3.1. Local IL-32 Expression at mRNA Level

Relative *IL32* expression was determined in patient-matched samples from tumor and non-transformed mucosa adjacent to tumor (52 pairs), obtained from 17 patients with ESCC, 14 with GC, and 21 with CRC using reverse-transcribed quantitative (real-time) polymerase chain reaction (RT-qPCR).

**Figure 1:**
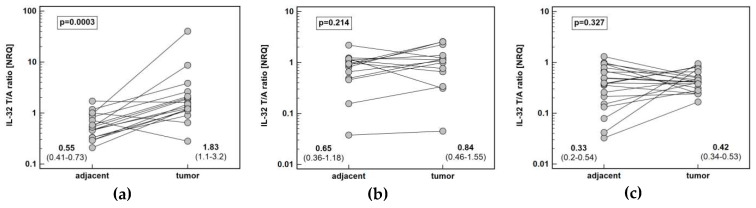
Pair-wise analysis of IL-32 expression at mRNA level: (**a**) in esophageal squamous cell carcinoma; (**b**) in gastric adenocarcinoma; (**c**) in colorectal adenocarcinoma. Data presented as geometric means of normalized relative quantities (NRQ) with 95% confidence interval and analyzed using *t*-test for paired samples.

**Figure 2:**
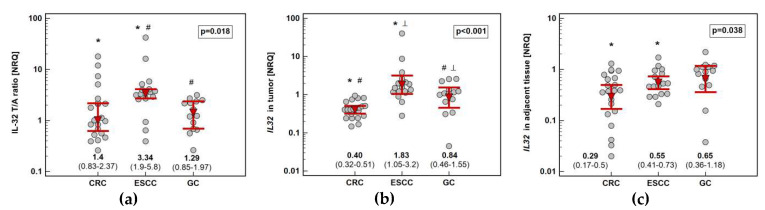
Impact of cancer type on *IL32* expression: (**a**) fold-change in expression in tumor and non-cancerous tumor-adjacent tissue (T/A ratio); (**b**) in tumors; (**c**) in non-cancerous tumor-adjacent tissue. Data presented as geometric means of normalized relative quantities (NRQ) with 95% confidence interval (red triangles with whiskers and numeric data below the dot-plots) and analyzed using one-way analysis of variance with Student–Newman–Keuls post-hoc test. Significant between-groups differences are marked by the symbols (*, #, ⟂) of the same type. CRC, colorectal cancer; ESCC, esophageal squamous cell carcinoma; GC, gastric cancer.

Pairwise analysis showed that *IL32* expression is significantly upregulated (by 3.3-fold) solely in esophageal tumors (Figure 1). Tumor expression of *IL32* was the highest in ESCC and the lowest in CRC. Esophageal tumors had higher interleukin expression by 2.2-fold as compared to gastric tumors and by 4.6-fold as compared to colorectal tumors. Gastric tumors had *IL32* expression higher than colorectal tumors by 2.1-fold. In the non-cancerous tumor-adjacent tissue, *IL32* expression was significantly higher in esophageal than colorectal mucosa (by 2.4-fold) and tended to be higher in gastric as compared to colorectal mucosa (by 2.3-fold). Consequently, fold-change in expression (tumor-to-adjacent) was comparable in CRC and GC and significantly lower than in ESCC by 2.4 and 2.6-fold, respectively (Figure 2).

There was no significant association between fold-change in *IL32* expression and cancer overall TNM stage or its individual components. Separate analysis conducted in tumors and non-cancerous adjacent tissue showed that *IL32* expression significantly increased along with growing depth of tumor invasion (T stage) in ESCC patients in adjacent tissue but not in tumors: (*ρ* = 0.51, *p* = 0.036).

Cardia subtype of GC (*n* = 5) tended to have higher expression of *IL32* in non-cancerous adjacent tissue than non-cardia GC (*n* = 9) by 2.1-fold (*p* = 0.115), while the transcript abundance in tumors was almost identical (*p* = 0.885). Consequently, fold-change in *IL32* expression tended to be higher in non-cardia GC by 1.9-fold (*p* = 0.124).

**Figure 3:**
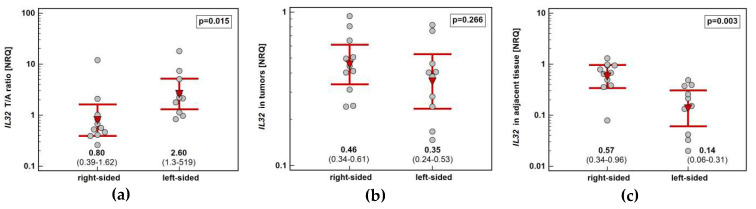
Impact of tumor location in the colon on *IL32* expression: (**a**) fold-change in expression in tumor and non-cancerous tumor-adjacent tissue (T/A ratio); (**b**) in tumors; (**c**) in non-cancerous tumor-adjacent tissue. Data presented as geometric means of normalized relative quantities (NRQ) with 95% confidence interval (red triangles with whiskers and numeric data below the dot-plots) and analyzed using *t*-test for independent samples.

Tumor location in the left side of the colon (*n* = 10) was associated with higher *IL32* upregulation (by 3.3-fold) than in the right side (*n* = 11). It resulted from significantly higher interleukin expression in non-cancerous tumor-adjacent mucosa from right than left side of the colon (by 4.1-fold) (Figure 3).

IL-32 has been implicated in modulating inflammation, immunity, proliferation, survival, angiogenesis, and epithelial-mesenchymal transition [15]. Therefore, the pattern of correlation between *IL32* expression and a broad spectrum of genes encoding representative proteins implicated in cancer development, namely, *IL4*, *IL4Ra*, *IL7*, *IL7Ra*, *IL10*, *IL10Ra*, *IL13*, *IL13Ra*, *ACTA2*, *BCL2*, *BCLxL*, *CCL2*, *CDKN1A*, *CLDN2*, *SLC2A1*, *HIF1A*, *Ki67*, *NOS2*, *ODC1*, *PTGS2*, *TJP1*, and *VEGFA*, was examined in 45 cancer patients.

In tumor tissue, *IL32* correlated positively with *ACTA2*, *BCL2*, *BCLxL*, *CCL2*, *HIF1A*, *IL13Ra*, *IL7*, *Ki67*, *ODC1*, *PTGS2*, *SLC2A1*, and *TJP1*. In non-cancerous tumor-adjacent tissue, *IL32* correlated positively with *BCL2*, *BCLxL*, *CCL2*, *CDKN1A, HIF1A*, *IL10Ra*, *IL13Ra*, *IL7Ra*, *Ki67*, *ODC1*, *SLC2A1*, *TJP1*, and *VEGFA*. Fold-change in *IL32* expression (tumor-to-adjacent) positively correlated with fold-change in expression of *BCLxL*, *CCL2*, *CDKN1A, HIF1A*, *IL7*, *IL7Ra*, *Ki67*, *SLC2A1*, and *VEGFA* (Table 2).

Multiple regression with genes significantly correlated with *IL32* in univariate analysis as explanatory variables showed *ACTA2* (r_p_ = 0.33, *p* = 0.043), *HIF1A* (r_p_ = 0.49, *p* = 0.002), *Ki67* (r_p_ = 0.46, *p* = 0.003), and *TJP1* (r_p_ = −0.48, *p* = 0.002) to be independent predictors of variation in *IL32* expression in tumors.

In adjacent tissue, of the genes associated with *IL32* expression in univariate analysis, *IL10Ra* (partial correlation coefficient: r_p_ = 0.59, *p* < 0.0001) and *SLC2A1* (r_p_ = 0.32, *p* = 0.041) were independently associated with the interleukin expression. Comparably well-fit regression model could be built with *BCLxL* (r_p_ = 0.64, *p* < 0.0001) instead of *SLC2A1*.

In turn, fold-change in *IL32* expression was independently associated with fold-change in expression of *IL7Ra* (r_p_ = 0.40, *p* = 0.008) and *SLC2A1* (r_p_ = 0.56, *p* < 0.001) or fold-change in expression of *CDKN1A* (r_p_ = 0.49, *p* = 0.001) and *IL7* (r_p_ = 0.43, *p* = 0.004), depending on regression model applied.

### 3.2. Local IL-32 Protein Concentration

The concentration of IL-32 (protein) was determined in homogenates of patient-matched tumor and non-cancerous tumor-adjacent tissue (63 pairs) from 17 ESCC patients, 12 GC patients and 34 CRC patients using dedicated immunoassays.

**Figure 4:**
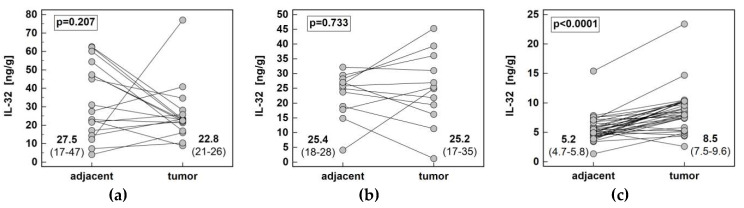
Pair-wise analysis of IL-32 concentration (protein) in tissue homogenates: (**a**) in esophageal squamous cell carcinoma; (**b**) in gastric adenocarcinoma; (**c**) in colorectal adenocarcinoma. Data presented as medians of interleukin concentration normalized to tissue weight with 95% confidence interval and analyzed using Wilcoxon test.

**Figure 5:**
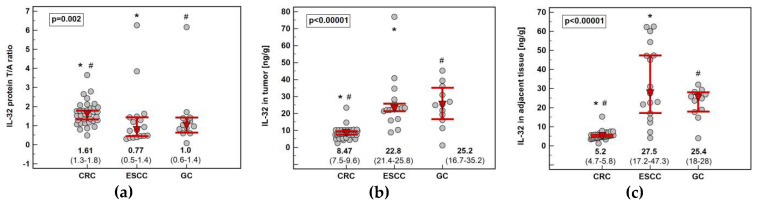
Impact of cancer type on IL-32 concentration (protein): (**a**) fold-change in concentration in tumor and non-cancerous tumor-adjacent tissue (T/A ratio); (**b**) in tumors; (**c**) in non-cancerous tumor-adjacent tissue. Data presented as medians with 95% confidence interval (red triangles with whiskers and numeric data below the dot-plots) and analyzed using Kruskal–Wallis *H* test with Conover post-hoc test. Significant between-groups differences are marked by the symbols (*, #) of the same type. CRC, colorectal cancer; ESCC, esophageal squamous cell carcinoma; GC, gastric cancer.

The interleukin concentration was significantly higher in tumor than adjacent tissue in CRC, showed no difference in GC and tended to be lower in ESCC (Figure 4). The IL-32 concentration in tumor (*p* < 0.00001) and adjacent tissue (*p* < 0.00001) as well as fold-change in concentration (tumor-to-adjacent) were significantly lower in CRC as compared to GC and ESCC (Figure 5).

**Figure 6:**
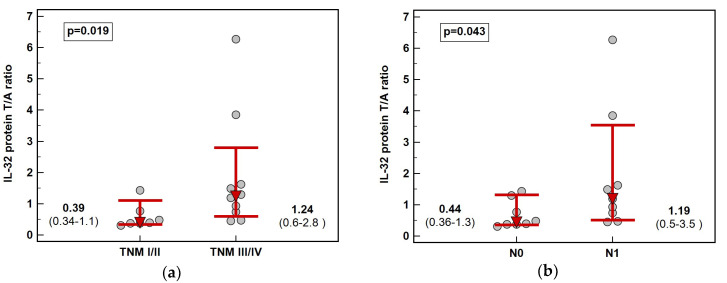
Impact of ESCC advancement on IL-32 protein concentration: (**a**) tumor-node-metastasis (TNM) stage; (**b**) lymph node involvement (stage N). Data presented as medians with 95% confidence interval (red triangles with whiskers and numeric data below the dot-plots). ESCC, esophageal squamous cell carcinoma.

Solely in ESCC, fold-change in IL-32 protein concentration (tumor-to-adjacent) was dependent on cancer stage. The interleukin was downregulated in tumors in early (TNM I and II) and slightly upregulated in advanced (TNM III and IV) cancers and the difference in fold-change was 3.2-fold (Figure 6). Significant difference was observed between N0 and N1 cancers (Figure 2) while the difference between T1/2 and T3/4 did not reach statistical significance (respectively, 0.4 and 1.2, *p* = 0.131).

Tumors of gastric cardia (*n* = 5) tended to have higher concentration of IL-32 than non-cardia tumors (*n* = 7) by 2.0-fold (*p* = 0.169), accompanied by slightly lower abundance in non-adjacent tissue (by 1.3-fold, *p* = 0.565). Fold-change in IL-32 protein concentration tended to be higher in cardia GC by 2.6-fold (*p* = 0.099).

**Figure 7:**
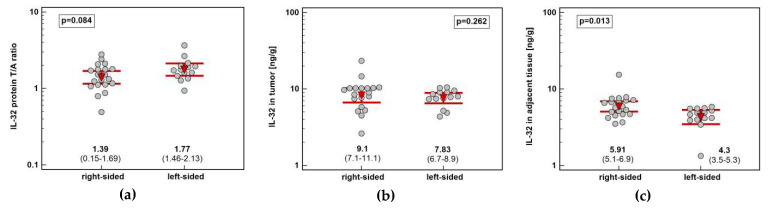
Impact of tumor location in the colon on IL-32 concentration: (**a**) fold-change in expression in tumor and non-cancerous tumor-adjacent tissue (T/A ratio); (**b**) in tumors; (**c**) in non-cancerous tumor-adjacent tissue. Data presented as geometric means with 95% confidence interval (red triangles with whiskers and numeric data below the dot-plots) and analyzed using *t*-test for independent samples.

Tumor location in the left side of the colon (*n* = 14) tended to be associated with greater IL-32 protein concentration than in the right side (*n* = 20) (by 1.3-fold). It resulted from significantly higher interleukin expression in non-cancerous tumor-adjacent mucosa from right than left side of the colon (by 1.4-fold) (Figure 7).

### 3.3. Systemic IL-32 Protein Concentration

The concentration of IL-32 (protein) was determined in serum samples from 17 ESCC patients, 12 GC patients and 41 CRC patients using dedicated immunoassays.

**Figure 8:**
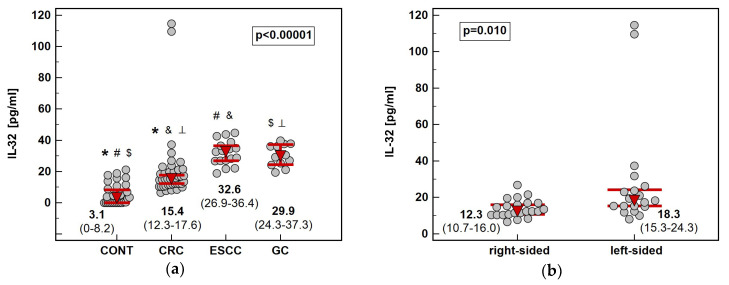
Circulating IL-32: (**a**) association with cancer type; (**b**) association with tumor location in the colon. Data presented as medians with 95% confidence interval (red triangles with whiskers and numeric data below the dot-plots) and analyzed using Kruskal–Wallis *H* test with Conover post-hoc test or Mann–Whitney *U* test. Significant between-group differences are marked with the same type of symbol (*, #, ⟂, $, &).

Cancer patients, regardless location, had significantly elevated circulating IL-32 as compared to healthy controls. Among cancers, CRC was accompanied by significantly lower IL-32 serum concentration than ESCC and GC. Tumor sublocation in the colon had an impact as well ‒ patients with left-sided tumors (*n* = 19) had significantly more elevated IL-32 than those with right-sided tumors (*n* = 22) (Figure 8). Regarding GC, cardia subtype (*n* = 5) was accompanied by insignificantly higher systemic IL-32 than non-cardia subtype (*n* = 7) (36 pg/mL vs. 29 pg/mL, *p* = 0.515).

Cancer stage had no significant effect on serum IL-32 in any cancer type.

Solely in GC, there was positive correlation between serum IL-32 and the local interleukin (protein) upregulation (r = 0.66, *p* = 0.020).

**Figure 9:**
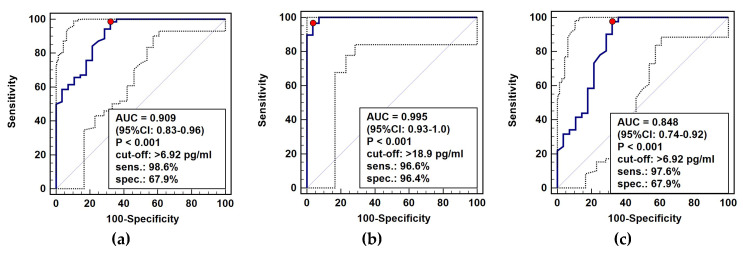
IL-32 as a cancer marker: (**a**) general cancer marker; (**b**) marker for gastroesophageal cancers (ESCC+GC vs. controls); (**c**) marker for colorectal cancer. Data presented as receiver operating (ROC) curves with 95% confidence interval (respectively, solid and dashed lines). AUC, area under ROC curve (indicating marker overall accuracy); CI, confidence interval; sens., sensitivity; spec., specificity accompanying optimal cut-off value (marked as a red dot); ESCC, esophageal squamous cell carcinoma; GC, gastric cancer.

The diagnostic power of circulating IL-32 in distinguishing cancer patients from healthy individuals was tested using receiver operating characteristic (ROC) curve analysis. As general cancer marker (all cancer patients analyzed against healthy controls), IL-32 was characterized by excellent accuracy and sensitivity but only fair specificity (Figure 9a). As a marker of gastroesophageal cancers (GC and ESCC against controls), the overall accuracy was near perfect and accompanied by excellent sensitivity and specificity (Figure 9b). For CRC, IL-32 was a marker of good accuracy and excellent sensitivity accompanied by fair specificity (Figure 9c).

For the purpose of correlation analysis we have retrieved data on systemic concentrations of IL-1β, IL-4, IL-6, IL-8, IL-12p70, FGF2, G-CSF, GM-CSF, MCP-1, MIP-1α, PDGF-BB, TNFα, and VEGF-A, available for 43 of our cancer patients. Circulating IL-32 correlated positively with G-CSF, PDGF-BB, and TNFα and negatively with FGF2, MIP-1α, and VEGF-A (Table 3).

## 4. Discussion

Despite growing interest in IL-32, there is a scarcity of data concerning its status in the GIT cancers while its diagnostic utility has not been previously determined. In line with a pro-inflammatory character of the interleukin and inflammation-related nature of investigated cancers [15], all patients had higher serum interleukin concentration than healthy individuals. While this finding corroborates previous observations regarding the GIT cancers [25,26,27,28], here we showed it to have a diagnostic significance. The interleukin displayed an excellent accuracy in discriminating between non-cancer controls and ESCC and GC patients, which was accompanied by very good sensitivity and specificity. This observation, if confirmed on larger cohort, is of utmost clinical importance, taking into account the significance of early cancer detection for patient prognosis. Especially that the diagnosis in GC and ESCC is delayed due to uncharacteristic symptoms and, in case of ESCC, a lack of clear precancerous stage [4]. The interleukin accuracy in detecting CRC was good, but inferior to ESCC and GC. Yet, at optimal cut-off, high sensitivity was accompanied by poor specificity. Worse performance could be explained by the fact that CRC patients in our cohort had significantly lower circulating IL-32 than those with ESCC and GC. Difference between cancers regarding interleukin concentration was notable also locally, although positive correlation between systemic and local IL-32 was found only in GC patients. Here, we showed that IL-32 in esophageal and gastric mucosa was expressed more markedly than in the colorectum, both at protein and mRNA level. Markedly higher interleukin expression was not limited to esophageal and gastric tumors but was notable in non-cancerous tumor-adjacent mucosa as well. The *IL32* transcripts were significantly overexpressed in tumors as compared to non-cancerous tissue only in ESCC patients. However, the observation was not corroborated at protein level, as IL-32 protein concentration was significantly upregulated solely in tumors from CRC patients and rather tended to be downregulated in ESCC. The discrepancy between the level of gene transcripts and protein is quite a frequent occurrence [29], previously observed for our patients with respect to expression of immunosuppressive IL-4 and IL-13 [22]. The phenomenon is explained by varying half-lives of mRNA and protein as well as by various posttranslational modifications which may further increase protein stability [29]. IL-32 expression has been repeatedly shown to be regulated by microRNAs [30,31], known to affect gene expression at transcriptional and/or translational level [32]. In fact, it has been suggested that transcriptomic and proteomic data should be treated as separate source of information [29]. In case of our study, the discrepancy might be associated with uncertainty of detected IL-32 isoforms. While the primer pair used in transcriptomic analysis covered all main variants, the specificity of antibody was not stated by the assay manufacturer but could be limited to some isoform.

If not for significantly higher protein content in esophageal than colonic mucosa, the discrepancy between ESCC and CRC could be attributed to differences in cancer histology. Accordingly, an analysis of IL-32 immunoreactivity in lung cancer has shown that adenocarcinomas had strong overexpression of interleukin-positive cells while the most of squamous cell carcinoma samples were lacking IL32-immunoreactivity [33]. Still, regarding the GIT cancers, IL32-positivity using the same antibody was higher in ESCC [34] than GC [35].

In an unpaired analysis, Yousif et al. [28] showed higher IL32-positivity in ESCC samples than non-cancerous mucosa obtained from patients operated for other reasons, which was also corroborated at mRNA level. We employed fully quantitative approach for IL-32 protein determination and used a paired design, analyzing patient-matched samples. While confirming Yousif’s et al. [28] results regarding mRNA, we failed to observe the IL-32 upregulation in tumors at protein level. As mentioned earlier, it may result from applied antibody being not optimal but may also indicate a cancer-associated accumulation of the interleukin in tumor-adjacent tissue. As has been repeatedly demonstrated [22,23,36,37,38], alterations in molecular profile of still non-transformed cells in tumor vicinity are common and may precede morphological and histological changes. The “tumor molecular margin” phenomenon predispose to neoplastic transformation and accounts for cancer recurrence and the synchronous tumors [39,40,41]. Supporting the notion, the abundance of *IL32* transcripts in non-cancerous tumor-adjacent esophageal mucosa increased along with growing depth of tumor invasion. An association between IL-32 and the T stage of ESCC was reported also by Nabeki et al. [34], although it concerned interleukin immunoreactivity. The authors have observed that high IL32-immunopositivity coincided with greater infiltration with regulatory T-cells, indicative of a more immunosuppressive environment. Here, fold-change in protein concentration between tumor and adjacent tissue only tended to be higher in locally advanced cancers but it was significantly associated with the presence of lymph node involvement and an overall ESCC stage, linking the upregulation of IL-32 protein in tumors with gaining metastatic potential.

The role of IL-32 gastric cancer seems to be unequivocally negative. It has been shown to be overexpressed in *H. pylori*-infected gastric mucosa and modulate cytokine synthesis [42]. In addition, it has been upregulated in gastric tumors and predictive of poor prognosis [35,43]. The IL32-immunopositivity has been associated with greater depth of tumor invasion, lymph node involvement, and venous invasion [35], which we failed to observe in our cohort, either at protein or mRNA level. However, like in our patients, no significant association with the disease advancement or patient outcome has been noted for serum IL-32 [26]. In turn, serum IL-32 has tended to be more elevated in cardiac than distal GC [26]. Even in a small set of samples in our study, we were able to observe a similar trend but regarding local interleukin expression. Mechanistically, IL-32 has been demonstrated to promote changes in gastric cancer cell morphology, facilitating their migration, and to enhance their potential for invasiveness by upregulating the expression of matrix metalloproteinases (MMP)-2 and 9, VEGF-A, and IL-8 via HIF-1α activation [19]. It has also been suggested that IL-32 may induce immunosuppression and allow cancer cells to evade immune system facilitating metastasis [35].

In turn, there seem to be a discrepancy concerning IL-32 status and role in CRC. Experimental findings mostly indicate a tumor suppressive function. The θ isoform of IL-32 (IL-32θ) inhibits migration by hampering epithelial-mesenchymal transition (EMT) and temper the properties of cancer stem cells [20]. IL-32α and γ mediate and enhance cell death induced by TNFα [44,45] by increasing production of reactive oxygen species [44] or by upregulating pro-apoptotic Bax [45] and reducing expression of anti-apoptotic Bcl-2 [45]. In animal models, IL-32α confers protection against azoxymethane-induced carcinogenesis [44] and IL-32γ [45] and IL-32β [46,47] suppress tumor growth. Mechanistically, it is associated with induction of apoptosis, downregulation of proliferation markers, reduced expression of proinflamatory enzymes and cytokines, and increased secretion of immunosuppressive *Il10* [46,47]. Contrary findings have been reported in humans. Catalan et al. [27] demonstrated IL-32α to be induced in response to hypoxia and to upregulate genes encoding mediators of inflammation and extracellular matrix remodeling. The CRC patients had elevated circulating IL-32 and their visceral adipose tissue had higher expression of the interleukin at both mRNA and protein level. Others have shown higher rates of IL32-immunopositivity in primary tumors derived from patients with lymph node metastasis as well as in metastatic as compared to corresponding primary tumors, regardless the location of distant metastases [48]. In our cohort, IL-32 has been significantly upregulated in colorectal tumors solely at protein level. Although not related to cancer pathology, its expression was dependent on the sublocation of primary tumors. Both *IL32* transcripts and IL-32 protein were more abundant in non-cancerous tumor-adjacent tissue from CRC patients with right-sided tumors, while interleukin tumor expression displayed a similar tendency. Consequently, the fold-change in expression ratio (tumor-to-adjacent) was higher in left-sided CRCs, significantly so for *IL32* transcripts. Circulating IL-32 was significantly more elevated in patients with tumors located in the left side of the colon. Although treated as one entity, CRC is highly heterogeneous with different set of protective and risk factors for tumors arising in right or left side of the colon and with subsite affecting disease presentation, treatment responsiveness and thus also patient prognosis. Right-sided tumors are considered to have less favorable characteristics than left-sided ones. Differences in clinical behavior stem from distinct genetic makeup and molecular patterns [49,50]. We and others have repeatedly documented dissimilarities between left- and right-sided cancers, not only locally [36,50,51,52,53] but also at systemic level [24,54,55,56] and now we showed the contribution of IL-32. This finding is of clinical relevance, since subsite heterogeneity, if not addressed, may hamper potential IL32-based therapies and reduce its diagnostic power as CRC biomarker.

To shed some light on possible role of IL-32 in GIT cancers, we analyzed the correlation patterns of the interleukin with major players in inflammation and immunity, cell proliferation and survival, angiogenesis and epithelial-mesenchymal transition, as well as metabolic reprogramming. Except for *ACTA2* and *PTGS2* (COX2) or *IL10Ra*, *IL7Ra*, and *VEGFA,* with which *IL32* correlated positively only in tumors or adjacent mucosa, respectively, the correlation patterns in cancerous and non-cancerous tissue were quite similar. Fold-change in *IL32* expression mirrored upregulation of proliferation marker *Ki67*, cell cycle regulator *CDKN1A* (p21^CIP1/WAF1^), metabolic reprogramming marker *SLC2A1*—encoding glucose transporter (GLUT1) angiogenic *VEGFA*, and *IL7* and its receptor *IL7Ra*. Multivariate analysis, allowing for discerning associations independent from other covariates, showed a tight positive relationship between tumor *IL32* expression and cell proliferation index and hypoxia, implying a tumor-promoting role for the interleukin. Mechanistically, the relationship between IL-32 and hypoxia can be bidirectional. Experimental studies in the colon have shown that cancer cell lines upregulate the interleukin expression in response to hypoxia while cell stimulation with IL-32α has no effect on *HIF1A* or *VEGFA* expression [27]. In the stomach, in turn, IL-32 have activated hypoxia-related transcriptional factor [19]. The interleukin, β and α, respectively, supported angiogenesis by upregulating VEGFA also in breast [45] and liver cancer cells [57]. Still, the proangiogenic effect of the interleukin on endothelial cells was independent from VEGF-A [58] what may explain lack of positive correlation between circulating IL-32 and VEGF-A. In fact, at the systemic level, the interleukin was inversely related with the growth factors as well as with FGF2, another potent proangiogenic factor, and a positive correlation was observed only between IL-32 and PDGF-BB. A negative relationship between IL-32 and VEGF-A has previously been reported in normal human bronchial epithelial cells [59]. An inverse relationship with FGF2 may be an echo of the inhibitory effect of the growth factor on fibroblast activation and mesenchymal transition [60]. It would be consistent with an EMT-promoting role ascribed to IL-32 overexpression, as discussed further.

Regarding proliferation, our findings oppose a role attributed to IL-32γ [46] and β [47] in the colon but are in agreement with the pro-proliferative activity of IL-32 in breast cancer [61]. When analyzed with reference to cancer type, *IL32* showed strong positive correlation with *Ki67* in gastric tumors (r = 0.72, *p* = 0.009) and only a weaker tendency in colonic tumors (*r* = 0.41. *p* = 0.072).

Tumor *IL32* expression in our clinical samples was independently associated also with EMT, positively with mesenchymal marker *ACTA2* (encoding α smooth muscle actin) and negatively with epithelial marker *TJP1* (encoding zona occludens-1), and thus indicative of interleukin involvement in promoting invasion and metastasis. Those results are consistent with experimental findings, showing IL-32α to upregulate expression of proteins engaged in remodeling of extracellular matrix (SPP1 and MMP9) in the colon [27] or other EMT markers, namely vimentin and Slug, in the liver [57]. Noteworthy, however, the θ isoform of the interleukin has reportedly a negative impact on EMT in the colon [20]. In the non-transformed tissue surrounding tumor, *IL32* expression was directly and independently associated with immunosuppressive and anti-inflammatory *IL10R*, consistent with the activity ascribed to IL-32β in the colon [47], or with antiapoptotic *BCLxL*. As others have shown that colonic cancer cells respond to IL-32 with induction of apoptosis [44], those results might indicate that IL-32 plays distinct roles at stages preceding neoplastic transformation than in already transformed cells.

In line with its role as an inflammation amplifier, the interleukin expression it is upregulated by TNFα in ESCC [62] and colon cancer [17] cell lines and, in a positive feed-back loop, activates NFκB in esophageal tumors [28] and upregulates TNFα expression and secretion in the colon [17,27]. Still, animal studies have shown IL-32γ and IL-32β to downregulate expression of inflammatory mediators [46,47]. Nonetheless, circulating interleukin is claimed to affect tumor microenvironment through positive regulation of pro-inflammatory cytokines [15] and to have modulatory effect on immune function [13,14]. Here, circulating IL-32 displayed a strong positive correlation with TNFα and G-CSF, while its local upregulation in tumors mirrored changes in the status of IL-7/IL-7Ra axis. There is no data linking IL-32 with G-CSF but GM-CSF, a colony-stimulating factor with overlapping functions in cancer, have been shown to induce expression of various isoforms of IL-32 in eosinophils [63]. The possible connection between IL-32 and IL-7/IL-7Ra axis is worth exploring in the light of increasing interest in this signaling pathway for anticancer therapy. Like IL-32, IL-7 in solid tumors is understudied and its role is poorly understood. Nonetheless, the accumulating evidence presents IL-7 as a cytokine facilitating tumor growth and metastasis and aiding drug-resistance [64].

A small number of available tissue samples, affecting the analysis in cancer subgroups, is a limitation that should be clearly acknowledged. Further studies on a larger cohort are needed to confirm the interleukin association with cancer pathology and tumor sublocation.

## Figures and Tables

**Table 1:** Characteristics of study population.

Characteristics	Controls	ESCC	GC	CRC	*p* Value
*n*	28	17	14	41	-
Sex (F/M), *n*	12/16	7/10	4/10	21/20	0.515 ^1^
Age [yrs.], median (95%*CI*)	57 (53–61.6)	61 (58–65)	64 (58–75.3)	59 (54–65.2)	0.121 ^2^
TNM stage (I/II/III/IV), *n*	-	1/6/9/1	2/3/5/4	14/12/12/4	0.079 ^1^
Primary tumor, T (1/2/3/4), *n*	-	0/6/9/2	1/1/8/4	5/11/22/3	0.201 ^1^
Lymph node metastasis, N (no/yes), *n*	-	8/9	5/9	26/15	0.159 ^1^
Distant metastasis, M (no/yes), *n*	-	16/1	10/4	37/4	0.118 ^1^

^1^ Chi-squared test; ^2^ Kruskal–Wallis H test. ESCC, esophageal squamous cell carcinoma; GC, gastric cancer; CRC, colorectal cancer; *n*, number of observations; F/M, female-to-male ratio; yrs., years; CI, confidence interval; TNM, tumor-node-metastasis cancer staging system.

**Table 2:** Correlation pattern of *IL32* and cancer-promoting genes.

Gene	Tumor *IL32*	Adjacent *IL32*	Fold-Change (T/A) in *IL32*
*ACTA2*	0.33, *p* = 0.031	ns	ns
*BCL2*	0.39, *p* = 0.019	0.39, *p* = 0.008	ns
*BCLxL*	0.34, *p* = 0.023	0.57, *p* < 0.001	0.37, *p* = 0.015
*CCL2*	0.45, *p* = 0.002	0.32, *p* = 0.037	0.30, *p* = 0.049
*CDKN1A*	0.27, *p* = 0.079	0.41, *p* = 0.006	0.51, *p* < 0.001
*HIF1A*	0.57, *p* < 0.001	0.40, *p* = 0.007	0.31, *p* = 0.040
*IL10*	0.29, *p* = 0.053	ns	ns
*IL10Ra*	ns	0.41, *p* = 0.005	ns
*IL13Ra*	0.37, *p* = 0.012	0.40, *p* = 0.007	ns
*IL7*	0.32, *p* = 0.037	0.40, *p* = 0.007	0.41, *p* = 0.005
*IL7Ra*	ns	0.37, *p* = 0.012	0.38, *p* = 0.011
*Ki67*	0.62, *p* < 0.001	0.49, *p* < 0.001	0.48, *p* = 0.001
*ODC1*	0.45, *p* = 0.002	0.35, *p* = 0.019	0.29, *p* = 0.056
*PTGS2*	0.30, *p* = 0.045	ns	ns
*SLC2A1*	0.54, *p* < 0.001	0.53, *p* < 0.001	0.55, *p* < 0.001
*TJP1*	0.39, *p* = 0.008	0.31, *p* = 0.041	ns
*VEGFA*	ns	0.52, *p* < 0.001	0.46, *p* = 0.002

Data analyzed following log-transformation and presented as Pearson correlation coefficient (*r*). T/A, tumor-adjacent ratio; ns, non-significant; *ACTA2*, smooth muscle actin alpha 2; *BCL2*, B-cell lymphoma 2; *BCLxL*, B-cell lymphoma-extra large; *CCL2*, C-C motif chemokine ligand 2; *CDKN1A*, p21^CIP1/WAF1^; *HIF1A*, hypoxia-inducible factor 1α; *IL10*, interleukin 10; *IL10Ra*, interleukin 10 receptor subunit α; *IL13Ra*, interleukin 13 receptor subunit α; *IL7*, interleukin 7; *IL7Ra*, interleukin 7 receptor subunit α; *Ki67*, proliferation marker Ki67; *ODC1*, ornithine decarboxylase 1; *PTGS2*, cyclooxygenase 2; *SLC2A1*; glucose transporter 1; *TJP1*; tight junction protein 1; *VEGFA*, vascular endothelial growth factor A.

**Table 3:** Correlation pattern of serum IL-32 and circulating cytokines and growth factors.

Cytokine/Growth Factor	Spearman Correlation Coefficient (*ρ*), *p*
FGF2	−0.52, *p* < 0.001
G-CSF	0.53, *p* < 0.001
MIP-1α	−0.35, *p* = 0.021
PDGF-BB	0.46, *p* = 0.002
TNFα	0.63, *p* < 0.001
VEGF-A	−0.52, *p* < 0.001

FGF2, fibroblast growth factor 2, G-CSF, granulocyte colony-stimulating growth factor; MIP-1α, macrophage inflammatory protein 1α; PDGF-BB, platelet-derived growth factor BB; TNFα, tumor necrosis factor α; VEGF-A, vascular endothelial growth factor A.

## Abbreviations

AUC	Area under the ROC curve
CI	Confidence interval
COX	Cyclooxygenase
CRC	Colorectal adenocarcinoma
EMT	Epithelial-mesenchymal transition
ESCC	Esophageal squamous cell carcinoma
FGF	Fibroblast growth factor
GC	Gastric adenocarcinoma
G-CSF	Granulocyte colony-stimulating factor
GIT	Gastrointestinal tract
GM-CSF	Granulocyte-macrophage colony-stimulating factor
IBD	Inflammatory bowel disease
IFN	Interferon
IL	Interleukin
MCP	Monocyte chemoattractant protein
MIP	Macrophage inflammatory protein
NOS	Nitric oxide synthase
NRQ	Normalized relative quantities
PDGF	Platelet-derived growth factor
ROC	Receiver operating characteristics
RT-qPCR	Reverse-transcribed quantitative (real-time) polymerase chain reaction
TNF	Tumor necrosis factor
TNM	Tumor-node-metastasis cancer staging system
VEGF	Vascular endothelial growth factor
